# Triggering necroptosis in cisplatin and IAP antagonist-resistant ovarian carcinoma

**DOI:** 10.1038/cddis.2014.448

**Published:** 2014-10-30

**Authors:** K E McCabe, K Bacos, D Lu, J R Delaney, J Axelrod, M D Potter, M Vamos, V Wong, N D P Cosford, R Xiang, D G Stupack

**Affiliations:** 1 Department of Reproductive Medicine, Moores Cancer Center, University of California, San Diego, La Jolla, CA, USA; 2 Nankai University School of Medicine, Tianjin, China; 3 Sanford-Burnham Medical Research Institute, La Jolla, CA, USA

## Abstract

Ovarian cancer patients are typically treated with carboplatin and paclitaxel, but suffer a high rate of relapse with recalcitrant disease. This challenge has fostered the development of novel approaches to treatment, including antagonists of the ‘inhibitor of apoptosis proteins' (IAPs), also called SMAC mimetics, as apoptosis-inducing agents whose action is opposed by caspase inhibitors. Surprisingly, IAP antagonist plus caspase inhibitor (IZ) treatment selectively induced a tumor necrosis factor-*α* (TNF*α*)-dependent death among several apoptosis-resistant cell lines and patient xenografts. The induction of necroptosis was common in ovarian cancer, with expression of catalytically active receptor-interacting protein kinase-3 (RIPK3) necessary for death, and in fact sufficient to compromise survival of RIPK3-negative, necroptosis-resistant ovarian cancer cells. The formation of a necrosome-like complex with a second critical effector, receptor-interacting serine–threonine kinase-1 (RIPK1), was observed. RIPK1, RIPK3 and TNF*α* were required for the induction of death, as agents that inhibit the function of any of these targets prevented cell death. Abundant RIPK3 transcript is common in serous ovarian cancers, suggesting that further evaluation and targeting of this RIPK3-dependent pathway may be of clinical benefit.

Comprising only 3% of all cancer cases in women in the United States, ovarian cancer is nonetheless the fifth leading cause of cancer-specific mortality.^1^ Approximately 90% of ovarian cancers are epithelial cancers derived from ovarian surface or fallopian tube epithelium.^2^ Serous ovarian carcinoma is the most common histologic subtype, with high-grade serous ovarian cancer (HGSOC) the most aggressive subtype, constituting 90% of these cases.^3^ Owing to its predominance and lethal nature, HGSOC is the most widely researched type of ovarian cancer.^3^

Typical treatment of HGSOC includes initial surgical debulking and subsequent systemic or intraperitoneal carboplatin and paclitaxel. While many tumors initially respond, 60–85% of patients experience disease recurrence following primary therapy.^1,3^ Relapse is often accompanied by disease that has acquired resistance to these drugs. One mechanism implicated in recurrence is the evasion of apoptosis, a form of programmed cell death whose loss represents an established hallmark of cancer.^4^ Exploiting alternative cell death pathways, including necroptosis (‘programmed necrosis'), may offer an alternative strategy to treat such recurrent disease.^5^

The cellular inhibitor of apoptosis proteins (c-IAP1 and c-IAP2) represent promising targets for therapy, as they are overexpressed in many cancers and have important roles in both apoptotic and necroptotic death pathways.^6^ Upon binding of tumor necrosis factor *α* (TNF*α*) to TNF*α* receptor 1, the adaptor protein TRADD (tumor necrosis factor receptor type 1-associated death domain protein) is recruited to the cytosolic death domain of TNF*α* receptor 1.^7^ This facilitates subsequent receptor-interacting protein kinase-1 (RIPK1)^8^ and TRAF2/5 (TNF receptor-associated factor 2/5) binding,^9^ which leads to cellular inhibitor of apoptosis protein 1/2 (c-IAP1/2) recruitment. The formation of this TNF*α*-induced membrane-associated complex, known as complex I, results in NF-*κ*B (nuclear factor-*κ*-light-chain-enhancer of activated B cells) activation, apoptosis or necroptosis, depending on the proceeding intracellular signaling events.

Ubiquitination of RIPK1 by the E3 ligase c-IAP1/2 activates the canonical NF-*κ*B pathway, resulting in cell survival.^10^ Conversely, the deubiquitination of RIPK1 or, alternatively, the lack of ubiquitination following IAP antagonist (IAPa or I)-induced degradation of c-IAP1/2 leads to the formation of a cytosolic complex that includes FADD (Fas-associated protein with death domain) and caspase-8. This triggers *apoptosis* if caspases are active (complex IIa), or receptor-interacting serine–threonine kinase-3 (RIPK3)-dependent *necroptosis* in the presence of caspase inhibitors (complex IIb; necrosome).^11,12^

IAPa will induce apoptosis in specific triple-negative breast or ovarian cancer cell lines,^13, 14, 15^ an observation that supports the launch of NCT01681368: *A Phase II trial of the IAP antagonist Birinipant for advanced ovarian, fallopian tube, and peritoneal cancer* (http://www.clinicaltrials.gov). In contrast, activation of TNF*α*-dependent necroptosis via IAP antagonism has been demonstrated in a colon cancer cell line,^16^ although not in other carcinomas.

The triggering of necroptotic signaling among ovarian cancer cells could have important therapeutic implications. Here, we demonstrate that IAP antagonism, in the presence of a caspase inhibitor, triggers necroptosis in apoptosis-resistant ovarian cancer cells, and, moreover, that patient-derived tumor cells display sensitivity to this treatment. Activation of this death pathway was dependent on the kinase activity of RIPK3, and required RIPK1, both of which we show are frequently expressed in serous ovarian cancer, as well as the cytokine TNF*α*. Notably, this death pathway occurred selectively in apoptosis-resistant cells. The results suggest a common functional necroptosis pathway that might be exploited in future therapeutic designs.

## Results

### Treatment with IAPa and the protease inhibitor zVAD induces death of RIPK3-expressing ovarian cancer cells

**Figure 1:**
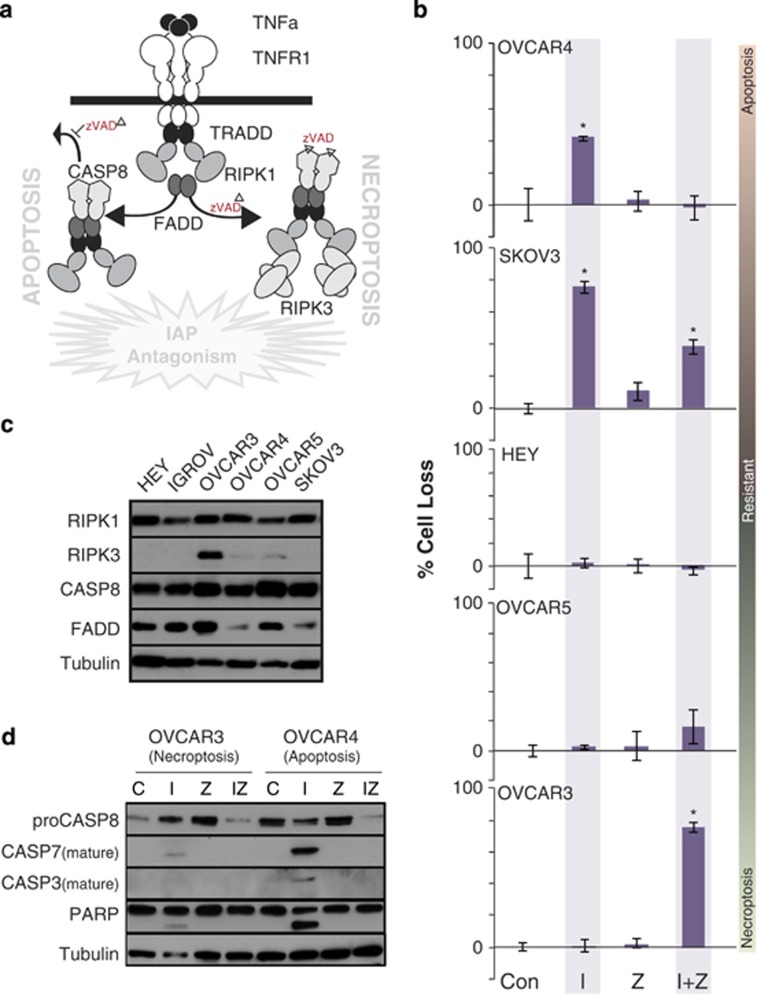
Activation of death pathways in ovarian cancer cells. (**a**) In the presence of IAP antagonism, TNF*α* receptor-mediated signaling can lead to apoptosis, or, in the presence of inhibitors of caspases such as zVAD that block apoptosis, a necrotic death triggered by RIPK1 and RIPK3. (**b**) Ovarian cancer cell lines treated for 48 h with diluent (Con), I (1 *μ*M), Z (20 *μ*M) or both (I+Z) were evaluated for cell loss (relative to Con). A representative experiment (of three or more for each cell line) is shown as mean of triplicates±S.D. *P<0.05 *versus* control. (**c**) The expression of proteins contributing to apoptosis or necroptosis was evaluated in ovarian cancer cells as indicated by immunoblot analysis. (**d**) Representative apoptotic (OVCAR4) and necroptotic (OVCAR3) cell lines were evaluated for poly-ADP ribose polymerase (PARP) cleavage and their capacity to elicit caspase maturation following 24 h treatment with I, Z or IZ as described in (**b**), above

A panel of human ovarian cancer cell lines were screened for the induction of apoptosis or necroptosis (as depicted in Figure 1a), following treatment with IAPa.^14^ Supporting the impetus to move IAPa forward in clinical trials, two of these (OVCAR4 and SKOV-3) showed some sensitivity to IAPa as a single agent (Figure 1b and He *et al.*^11^). This was at least partially blocked by the inhibitor of apoptosis z-VAD-fmk (pancaspase inhibitor, Z-Val-Ala-DL-Asp-FMK, Z) IAPa plus zVAD; IZ, which selectively, but not exclusively, inhibits caspase activity. Importantly, and perhaps not surprisingly, the majority of tumor lines tested showed resistance to the induction of apoptosis by either IAPa (Figure 1b: Hey, OVCAR5 and OVCAR3; not shown: IGROV1) or cisplatin (Supplementary Figure 1a). Neither caspase-8 nor FADD appeared to be limiting for apoptosis (Figure 1c). Indeed, the cells with the lowest expression of FADD were the most sensitive to the induction of apoptosis by IAP antagonism.

Interestingly, zVAD treatment actually promoted, rather than rescued, death in some cell lines (Figure 1b, bottom panels). This raised the possibility of the induction of an alternative form of programmed cell death: necroptosis. This notion was bolstered by the observation that apoptosis-resistant but IAP antagonist plus caspase inhibitor (IZ)-sensitive lines exhibited expression of RIPK3 (Figures 1b and c), a critical regulator of necrotic cell death.^11^ Further supporting this possibility, cell death induced by IZ was not accompanied by the activation of caspases, as occurs during apoptosis (Figure 1d).^6^ As the concept that tumor cells (in particular serous ovarian tumor cells) might be sensitive to necroptosis had not been previously explored, we characterized this cell death further.

### Formation of the necrosome in IZ-sensitive cells

**Figure 2:**
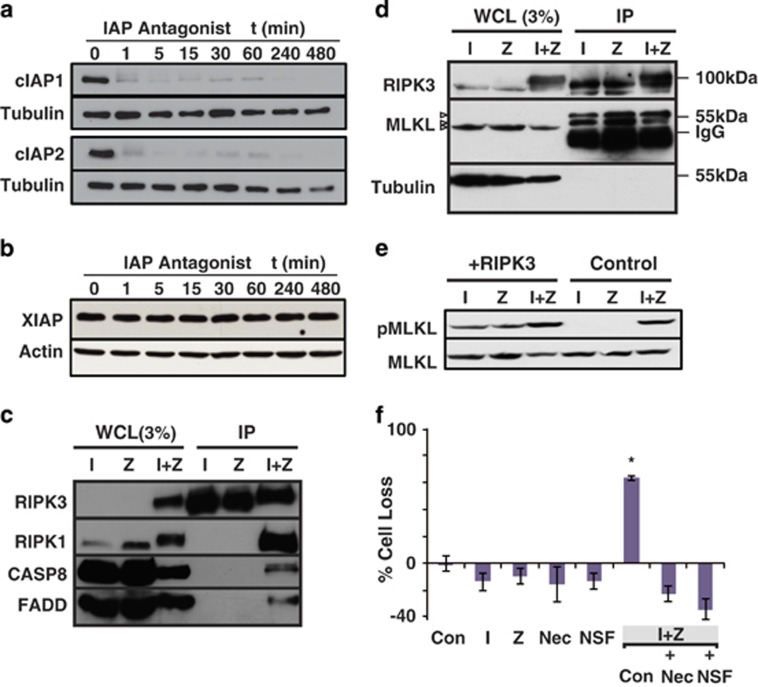
Evaluation of the necroptotic phenotype in ovarian cancer. Time course showing the effect of incubation of I (1 *μ*M) on (**a**) cIAP levels and (**b**) XIAP levels among ovarian tumor cells (OVCAR3) that exhibit a ‘necroptotic phenotype'. Actin was used as a loading control as XIAP and tubulin have a similar mass. (**c**) Immunoprecipitation of a complex containing RIPK1 and RIPK3, as well as FADD and caspase-8, following treatment of OVCAR3 cells expressing mCherry-tagged RIPK3 with either I (1 *μ*M), Z (20 *μ*M) or both (I+Z) for 24 h. (**d**) Formation of a RIPK3 complex containing MLKL was evaluated by immunoblotting the mCherry immunoprecipitates described. The MLKL-reactive species are shown as open arrowheads. (**e**) Evaluation of MLKL phosphorylation in OVCAR3 cells or OVCAR3 cells expressing ectopic RIPK3 as a positive control. Cells were again treated with I, Z or I+Z as detailed above, and expression of pMLKL or total MLKL was evaluated by immunoblot analysis. (**f**) Effect of I, Z, necrostatin (Nec, 10 *μ*M) or necrosulfonamide (2 *μ*M) treatment, alone or added in combinations as shown, for 48 h, on ovarian cancer cell death. Mean±S.D. of triplicates from representative experiments are shown. *P<0.05 *versus* control

It remained possible that necrosis occurred as a default pathway when IAPa were ineffective at inducing the clearance of IAPs required for apoptosis.^13^ To test this, we first evaluated the presence of two IAPs (c-IAP1 and c-IAP2) following antagonist treatment (Figure 2a). As shown, IAPa treatment resulted in the complete and persistent loss of cIAPs within minutes. Thus, IAP loss is consistent with necroptotic death. However, a general loss of all IAPs was not observed, as treatment did not appear to influence the expression of X-linked inhibitor of apoptosis (XIAP) (Figure 2b). A third target of IAPa, ML-IAP (a member of IAP family, containing a single copy of a baculovirus IAP repeat (BIR) as well as a RING-type zinc-finger domain), was not expressed in these cells (Supplementary Figure 1b).

To evaluate whether a functional necrosome complex was indeed forming, we next immunoprecipitated RIPK3 expressed in the ovarian cancer cells and tested for the presence of associated proteins. IZ treatment resulted in the formation of a complex with abundant representation of RIPK1, but with much lower levels of FADD and caspase-8 (Figure 2c). Treatment with either agent alone (I or Z) did not result in the formation of a complex (Figure 2c). In contrast, mixed lineage kinase domain-like (MLKL) was constitutively associated with RIPK3 under all three conditions (Figure 2d). As formation of the necrosome promotes the phosphorylation of MLKL as a downstream effector of RIPK3, we next confirmed that IZ treatment did, in fact, result in marked phosphorylation of MLKL (Figure 2e). In fact, simple ectopic expression of RIPK3 led to a lower basal level of MLKL phosphorylation, although this was insufficient to induce cell death.

Further supporting a model of classical RIPK1-dependent necroptosis, IZ-induced death was rescued by the inclusion of necrostatin-1, a RIPK1 inhibitor^17^ (Figure 2f). Cell death was alternatively also blocked by inclusion of necrosulfonamide (Figure 2f), which targets the death-inducing RIPK3 substrate, MLKL.^18^ These results, together, suggest that necroptosis is induced by activation of RIPK1 followed by activation of RIPK3 and MLKL in ovarian cancer cells.

### RIPK1 expression is required for IZ-induced death of ovarian cancer cells

**Figure 3:**
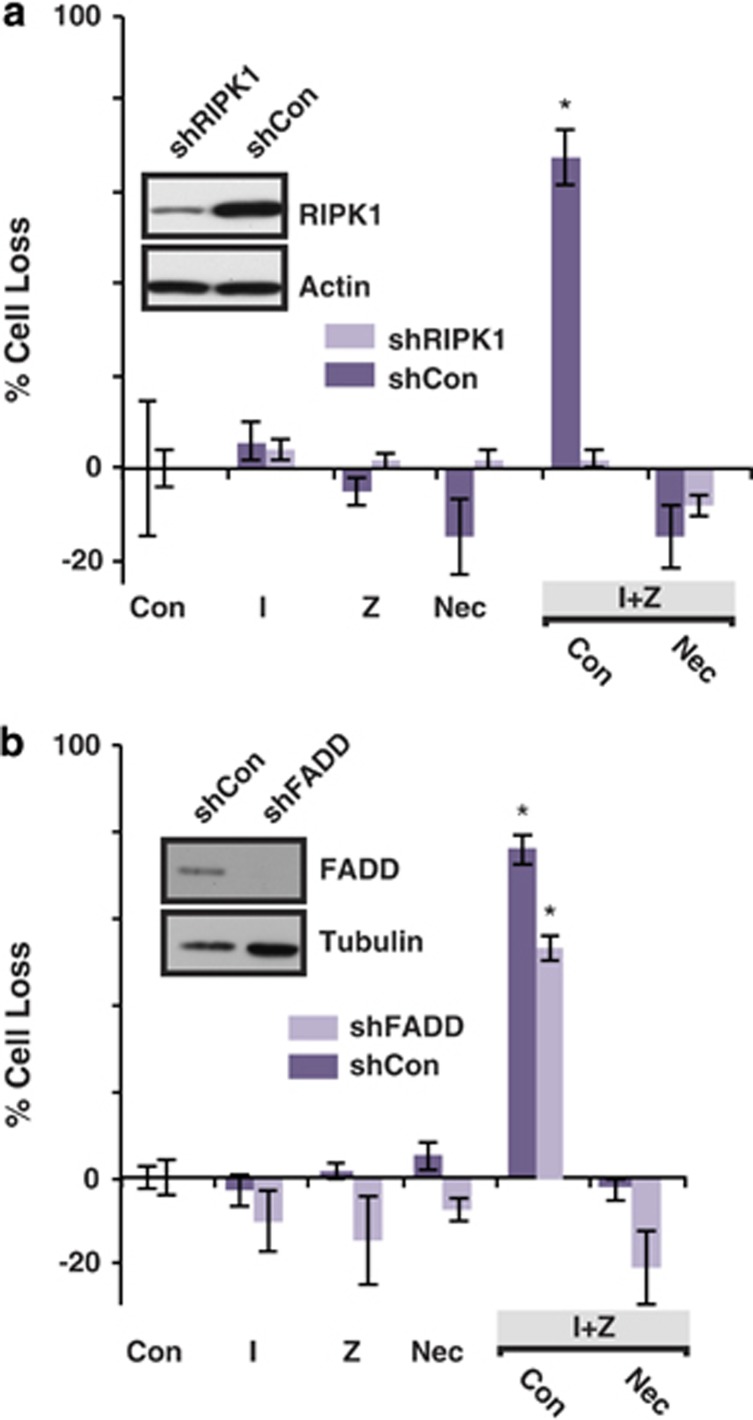
RIPK1 suppression compromises I+Z-mediated death in ovarian cancer cells. (**a**) The effect of suppression of RIPK1 expression by shRNA (see inset) on OVCAR3 cell death was examined following treatment with I (1 *μ*M), Z (20 *μ*M), necrostatin (Nec), diluent controls (Con) or combinations of these agents as shown for 48 h. (**b**) The effect of suppression of the adaptor protein FADD (inset) was similarly evaluated. Mean±S.D. of triplicates from representative experiments are shown. *P<0.05 *versus* control

All of the ovarian cancer tumor cell lines examined expressed RIPK1, and its expression was not sufficient for, or predictive of, cell death following I or IZ treatment (Figure 1b). As necrostatin-1 inhibited cell death, we next silenced RIPK1 expression (Figure 3a, inset) and then evaluated cell survival following exposure to IZ. As shown, the loss of RIPK1 was protective, consistent with the necrostatin sensitivity of the death pathway (Figure 3a). By contrast, the knockdown of the adaptor protein FADD had a more modest effect (Figure 3b), suggesting that FADD may provide some facilitating role in necroptosis in the ovarian cancer cells, yet was not critical in facilitating RIPK1 to RIPK3 signaling.

### RIPK3, but not an inactive mutant, promotes IZ-induced death

**Figure 4:**
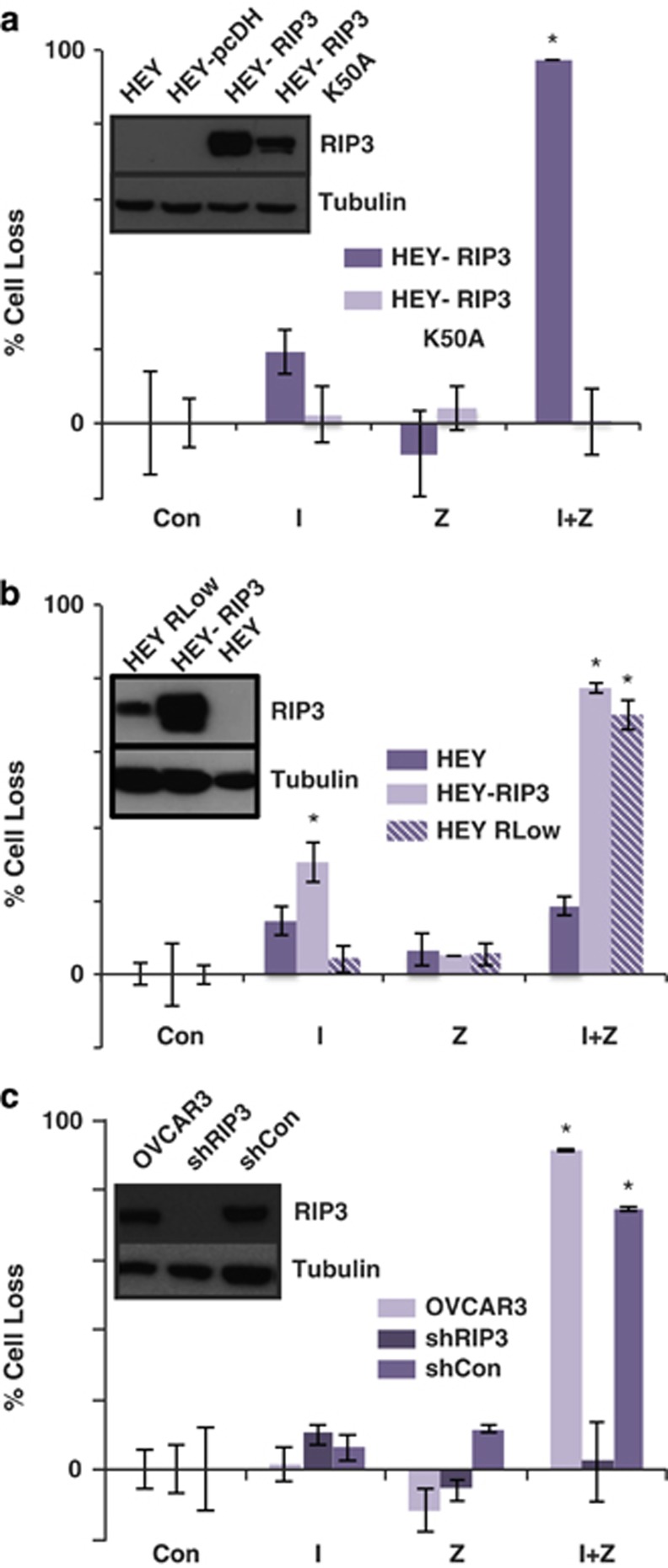
RIPK3 expression is required to trigger cell death after I+Z treatment. (**a**) The effect of reconstitution of expression of RIPK3, or a catalytically dead mutant (K50A), in the death-resistant HEY cells was assessed for impact on cell death 48 h following treatment with I (1 *μ*M), Z (10 *μ*M), necrostatin (Nec, 10 *μ*M), diluent controls (Con) or combinations of these agents as shown. (**b**) Expression of low levels of RIPK3 was also evaluated for impact on cell death, as described above. (**c**) The effect of suppression of RIPK3 by shRNA (inset) on OVCAR3 cell death was examined following treatment with I (1 *μ*M), Z (10 *μ*M), diluent control or combinations of these agents as shown. Mean±S.D. of triplicates from representative experiments are shown. *P<0.05 *versus* control

To more carefully characterize the requirement for RIPK3, we next used genetic approaches to reconstitute RIPK3 expression in the RIPK-deficient, death-resistant HEY cells (Figures 1b and c) and to suppress RIPK3 expression in the death-inducible OVCAR3 cells. In agreement with the capacity of necrostatin to block IZ-induced death, the expression of RIPK3, but not a kinase dead RIPK3 mutant, was sufficient to confer sensitivity to IZ-induced death among HEY cells (Figure 4a). The expression level of the kinase dead version was, however, somewhat lower than that of the wild-type kinase, possibly due to other recently described death-inducing effects of catalytically compromised RIPK3.^19^ Selection of clones of HEY with much lower expression of RIPK3 similarly resulted in cell death following IZ treatment (Figure 4b), suggesting that relatively low levels of RIPK3 may be sufficient to confer sensitivity. In agreement with these observations, the loss of RIPK3 expression compromised cell death in IZ-sensitive cell lines (Figure 4c). Thus, RIPK3 expression is a prerequisite for this cellular death pathway.

### IZ treatment promotes autocrine TNF*α* production

**Figure 5:**
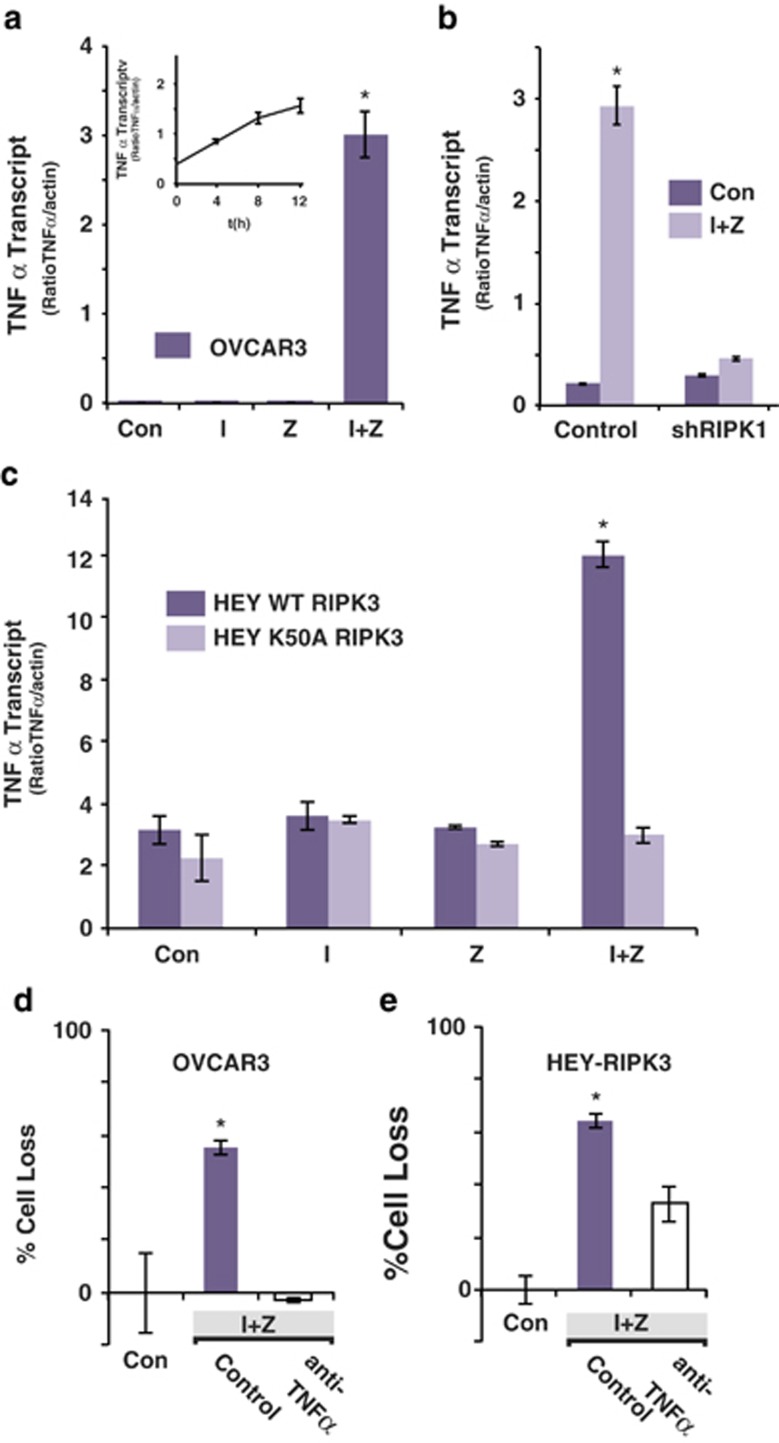
TNF*α* transcription is induced by I+Z treatment. (**a**) The relative level of TNF*α* transcript was evaluated by qPCR in diluent-treated OVCAR3 cells or following treatment with I (1 *μ*M), Z (20 *μ*M) or both. (**b**) Similar evaluation in OVCAR3 cells expressing shRNA to RIPK1 or a control shRNA, or in (**c**) HEY cells reconstituted with RIPK3 or a catalytically dead mutant (K50A) RIPK3. The influence of treatment anti-TNF*α* (5 *μ*g/ml) on I+Z-induced cell death was evaluated in the OVCAR3 cells (**d**) or HEY cells reconstituted with RIPK3 (**e**). Mean±S.D. of triplicates from representative experiments are shown. **a**–**c**: *P<0.05 *versus* control; **d** and **e**: *P<0.05 for I+Z control *versus* either untreated control or anti-TNF*α*

The TNF*α* receptor I must be activated by ligation and subsequent formation of the membrane-associated complex I (depicted in Figure 1a) for the necrosome to form and lead to necrotic cell death. We reasoned that without exogenous death receptor ligand, IZ-sensitive ovarian cancer cells might have to produce their own extrinsic death pathway stimulus. The relative TNF*α* mRNA levels in I- or Z-only treated cells was comparable to levels in vehicle-treated cells; however, a marked upregulation of TNF*α* was observed in IZ-treated OVCAR3 cells (Figure 5a), which was dependent on the expression of RIPK1 (Figure 5b). Ectopic expression of RIPK3 rescued IZ-induced TNF*α* in HEY cells (Figure 5c), suggesting that RIPK3 expression may enhance autocrine TNF*α* production by the ovarian cancer cells as it does in myeloid cells.^20^ The kinase activity of RIPK3 was critical for this effect, as the expression of catalytically dead RIPK3 (K50A) did not induced the expression of TNF*α* (Figure 5c). TNF*α* appeared to be required for death, as a TNF*α-*neutralizing antibody was able to rescue cell survival in ovarian carcinomas expressing endogenous (Figure 5d) or ectopic (Figure 5e) RIPK3.

### RIPK3 expression in ovarian cancer

**Figure 6:**
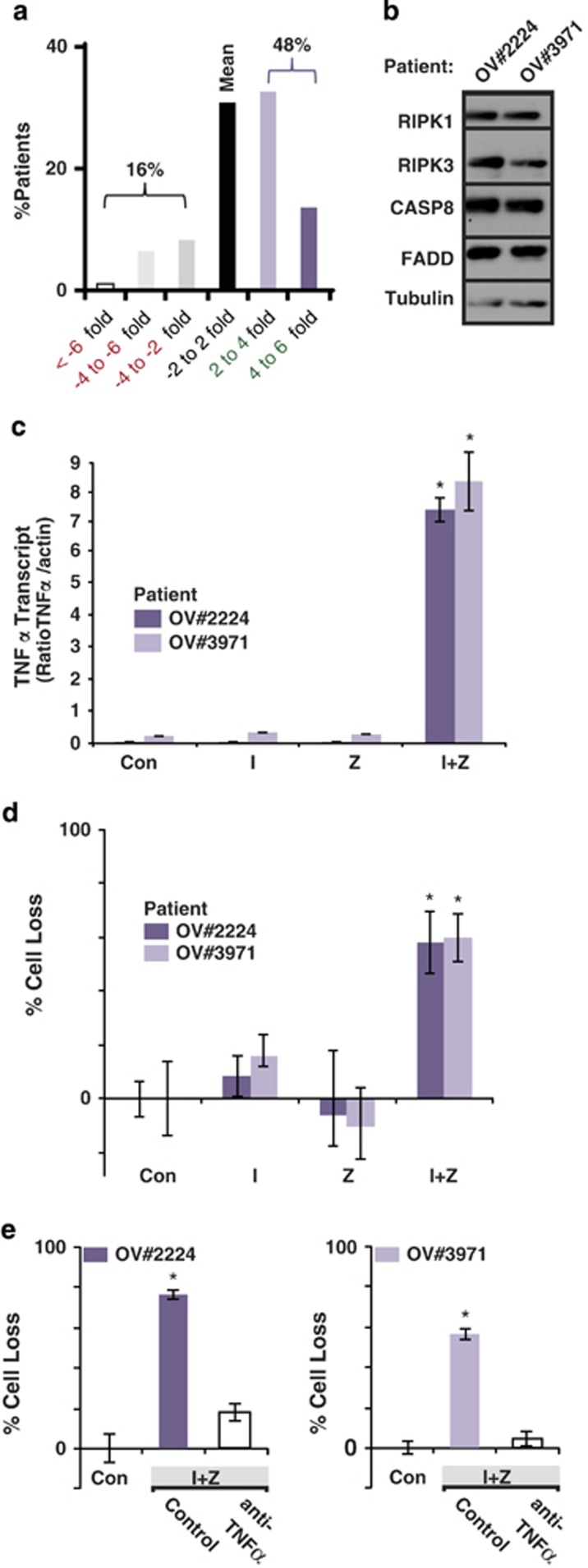
Expression of ripoptotic effectors in ovarian cancer. (**a**) The distribution of RIPK3 transcripts across all aggressive serous ovarian cancers curated in the TCGA web portal. (**b**) Immunoblot analysis for key effectors of apoptosis/necroptosis in xenograft tumor cells lysates derived from ovarian cancer patients. (**c**) TNF*α* transcript was evaluated by qPCR in patient-derived serous ovarian cancer cells following treatment with diluent (Con), I (1 *μ*M), Z (10 *μ*M) or both agents. (**d**) Patient-derived ovarian cancer cells were treated for 48 h with diluent (Con), I (1 *μ*M), Z (20 *μ*M) or both agents (I+Z) and evaluated cell death. (**e**) The impact of treatment with nonspecific Ig or anti-TNF*α* (5 *μ*g/ml) on IZ-induced cell death of patient-derived ovarian cancer cells. Mean±S.D. from one of two similar studies are shown. **c** and **d**: *P<0.05 *versus* control; **e** and **f**: *P<0.05 I+Z for control *versus* either untreated control or anti-TNF*α*

Although RIPK3 expression promotes necroptosis, we only noted the expression of RIPK3 in a few ovarian cancer cell lines. This raised the question as to what the relative expression of RIPK3 in ovarian tumors might be. We focused on serous ovarian cancer, which is well curated in the The Cancer Genome Atlas repository and also accounts for the greatest overall mortality among ovarian carcinoma patients. The database cataloged an enrichment for RIPK3 expression in serous ovarian cancer (Figure 6a). In fact, more than three-quarters of serous ovarian tumors expressed RIPK3 levels above the mean expression established across all tumors. Almost half of tumors were enriched at least twofold over the mean expression, as opposed to only one in six tumors, which was twofold or less below the mean (Figure 6a). As we had previously observed in the OVCAR3 cells, and consistent with the TCGA data set, our two cisplatin-resistant patient-derived tumor cells (OV no. 2224 and OV no. 3971) revealed RIPK3 protein expression upon immunoblot analysis (Figure 6b). Moreover, the patient-derived tumor cells demonstrated IZ-induced autocrine expression of TNF*α* (Figure 6c). Accordingly, we observed no induction of apoptosis with IAP antagonism alone, but IZ-induced cell death (Figure 6d) that was blocked by TNF*α*-neutralizing antibody (Figures 6e and f). Altogether, the results support a model in which necroptotic cell death is common following IAP antagonism.

## Discussion

The poor prognosis for patients with advanced ovarian cancer, combined with the universal loss of tumor protein p53 (TP53) in an otherwise molecular heterogeneous background, has led to increasingly innovative approaches to treat the disease. Although 70–80% of disease will respond to initial chemotherapy, recurrence of recalcitrant disease is common. Interestingly, the IZ-sensitive patient tumor cells used here also exhibited quite high cisplatin resistance (half-maximal inhibitory concentration >10 *μ*M). This observation in particular would appear to support the development of agents, such as Birinipant, that antagonize IAPs as adjuvants to existing therapies to prevent the selection of cisplatin resistance in tumors.

We examined several ovarian cell lines for their ability to undergo death following IAPa treatment. This was important, as attention has recently focused on the suitability of commonly used cell lines as models for serous ovarian cancer. Domcke *et al.*^1^ assessed the genetic similarity between commonly used ovarian cancer cell lines and serous ovarian tumor tissue samples to determine the likelihood of each cell line being of HGSOC origin. Interestingly, while the OVCAR3 cell line we study here was found to be similar to the general tumor tissue signature in this study, the other ovarian cancer cell lines that remain part of the NCI-60, such as HEY and SKOV-3, were not. Perhaps more importantly, the molecular signature of cell death proteins and the response to antagonism revealed that OVCAR3 cells were quite similar to patient-derived tumor cells typified by OV#2224 and OV#3871, and thus acted as a representative cell line for this system. Indeed, the cisplatin-resistant OVCAR3 may represent a preferred cell line for the analysis of emerging ovarian cancer treatments.

We demonstrate that TNF*α* production is required to trigger necroptosis in the ovarian cancer cells, and that RIPK3 enhanced this process. Consistent with recent reports, depletion of RIPK3 has a marked impact on the induction of necrosis,^16^ and notably, the expression of RIPK3 was sufficient to restore sensitivity to necroptosis in the apoptosis and necroptosis-resistant HEY cells. This required the production of TNF*α*, as antibodies to TNF*α* prevented IZ-mediated killing. Nonetheless, the IZ combination elicits additional effects, as simple addition of TNF*α* to either I or Z alone was not sufficient to promote cell death (KM, DGS, unpublished observations).

In these studies, the source of TNF*α* was autocrine. However, caution is warranted in overinterpreting this requirement *in vivo*, as TNF*α* can be produced by stromal or inflammatory cells in the tumor microenvironment.^21^ In this case, the *in vivo* use of IAPa that are thought to induce effectively apoptosis in patients might rather be effecting cell death by necroptosis. The requirements for, and the form of death may be important to understand in the clinical setting. In fact, understanding the type of death induced by an agent *in vivo* is more than just a semantic distinction. Increasingly, molecular determinants are used for the stratification of patients into preferred treatment groups. This is progressing rapidly, and the initial single gene requirements for patient stratification^22^ are destined to become a thing of the past, yielding to more complex algorithms for personalized medicine.^23^ Thus, the question raised by the current studies, that is, whether tumor cells undergo apoptosis or necroptosis *in vivo* following exposure to IAPs, becomes a key factor for the implementation of therapy, and should be addressed in future directed studies.

## Materials and Methods

### Reagents

IAPa were synthesized by Mitchell Vamos (Sanford-Burnham Medical Research Institute, La Jolla, CA, USA). The pancaspase inhibitor zVAD-fmk was purchased from Bachem (Torrance, CA, USA) (N-1510.0005BA). Necrostatin-1 (2324/10) and necrosulfonamide (432531-71-0) were purchased from R&D Systems (Minneapolis, MN, USA) and EMD Millipore (Temecula, CA, USA), respectively. The TNF*α-*neutralizing antibody was purchased from Cell Signaling Technology (Danvers, MA, USA; 7321). The following primary antibodies were used for western blotting: c-IAP1 (Cell Signaling Technology; 7065), c-IAP2 (Cell Signaling Technology; 3130), RIPK3 (Cell Signaling Technology; 12107), RIPK1 (Cell Signaling Technology; 3493), caspase-8 (BD Biosciences; 551242), FADD (BD Biosciences; 610399), p62 (BD Biosciences; 610832), LC3 (Novus Biologicals, Littleton, CO, USA; NB100-2220), *β*-actin (Sigma, St. Louis, MO, USA; A5441), PARP (BD Biosciences; 51-6639GR), caspase-3 (Cell Signaling Technology; 9668), caspase-7 (Cell Signaling Technology; 9492), XIAP (Cell Signaling Technology; 5471), MLKL (EMD Millipore; MAB C604), phospho-MLKL (Abcam, Vancouver, BC, USA; Ab187091) and *α*-tubulin (EMD Millipore; CP06). The following horseradish peroxidase-conjugated secondary antibodies were used for western blotting: goat anti-rabbit IgG (H+L) (Jackson ImmunoResearch, Bar Habor, ME, USA; 111-035-003), goat anti-mouse IgG (H+L) (Jackson ImmunoResearch; 115-035-003), goat anti-rabbit IgG, Fc fragment-specific (Jackson ImmunoResearch; 111-035-008) and mouse anti-rabbit IgG, light-chain-specific (Jackson ImmunoResearch; 211-032-171). The anti-mCherry antibody used for immunoprecipitation was generated in-house by immunization of rabbits with full-length mCherry and immunoaffinity purification of immune serum on a Sephadex-mCherry column.

### Cell culture

OV no. 2224 and OV no. 3971 cells were isolated from ovarian cancer patients, subcultured^12^ and passaged through mice. Informed consent was obtained to use these cells for research purposes. The human ovarian cancer cell lines HEY, OVCAR3, OVCAR4, OVCAR5 and SKOV-3 were maintained in no-glucose RPMI-1640 (Mediatech Inc., Manassas, VA, USA) supplemented with 1 g/l glucose, 10% FBS, MEM nonessential amino acids and 1 mM sodium pyruvate (Mediatech Inc.). All cells were maintained in an incubator set to 37 °C and 5% CO_2_.

### Generation of stable cell lines

All stable cell lines were generated by using lentiviral-based RNAi or lentiviral-based gene expression systems, as described previously.^24^ Briefly, shRIPK3 and shRIPK1 cell lines were selected for with 1 *μ*g/ml puromycin after being infected with lentivirus containing shRNA constructs specifically targeting RIPK3 or RIPK1 mRNA. Cell lines (OVCAR3 and HEY) stably expressing mCherry-tagged RIPK3 or a kinase dead mutant (K50A) were selected with 1 *μ*g/ml puromycin following lentivirus treatment with a pLV RIPK3-mCherry plasmid.

### IAP antagonism time-course assay

Cells were seeded into the wells of 6-well plates (2 × 10^6^ cells/well) and allowed to adhere for ~16 h at 37 °C before the addition of drug(s). Media were replaced with fresh media containing 1 *μ*M IAPa and cells were incubated at 37 °C for various amounts of time. At selected time points, media were aspirated and cells were washed two times with PBS and lysed with 150 *μ*l RIPA lysis buffer supplemented with a protease inhibitor cocktail (Sigma), 2 mM sodium orthovanadate and 50 mM NaF. Cells were collected using a cell lifter (Thermo Fisher, Waltham, MA, USA), transferred to an Eppendorf tube, passed through a pipet tip five times and incubated on ice for 10 min. The lysate was centrifuged at 14 000 × *g* for 5 min at 4 °C, and the supernatant (lysate) was collected and stored at −20 °C until western blot analysis.

### Growth inhibition assay

Cells were seeded into wells of a 96-well plate (10^4^ cells/well; 100 *μ*l final volume) and allowed to adhere for ~16 h at 37 °C before the addition of drug(s). Treatment or vehicle was added in a total dosage volume of 11 *μ*l (to final concentrations of 10 *μ*M necrostatin, 2 *μ*M necrosulfonamide, 20 *μ*M zVAD-fmk or 1 *μ*M IAPa). Cells were incubated at 37 °C for 48 h, washed gently two times with 100 *μ*l PBS and stained with 75 *μ*l crystal violet (Sigma) aqueous dye solution (0.1% crystal violet, 150 mM NaCl, 20% methanol) for 25 min  at room temperature. Stain was removed and cells were washed two times with water to remove unbound dye, and then allowed to dry. Cell-bound crystal violet was resolubilized with 75 *μ*l methanol and *A*_600_ was measured using a PowerWave XS2 Microplate Spectrophotometer (BioTek). All treatment conditions were assayed in triplicate and plotted as mean±S.D. Except as noted, all experiments were performed three or more times with similar results. ‘Cell loss' was calculated using the formula: 100−((*A*_600_ treatment/*A*_600_ vehicle) × 100).

### Immunoprecipitation

For the immunoprecipitation of mCherry-tagged RIPK3, cells were lysed with ‘NP-40 Alternative Lysis' (NAL) buffer (EMD Millipore; 492016) buffer (100 mM Tris, pH 8.0, 150 mM NaCl, 1% NP-40 Alternative) following 24 h exposure to the indicated agents. Cell mixtures were centrifuged at 2500 × *g* for 3 min, then total protein concentration was determined as described and 1 mg protein was diluted with lysis buffer to a final volume of 1 ml. Lysates were incubated with 5 *μ*g rabbit anti-mCherry antibody for ~16 h at 4 °C, followed by 20 *μ*l Protein A/G resin (G-Biosciences, St. Louis, MO, USA) for ~3 h at 4 °C. Following centrifugation at 2500 × *g* for 3 min, resin was washed four times with NP-40 Alternative wash buffer (20 mM Tris, pH 7.4, 150 mM NaCl, 0.1% NP-40 Alternative). Immunoprecipitates were eluted by heating in reducing sample buffer for 5 min at 100 °C, cooled to room temperature and briefly centrifuged to pellet beads. Supernatants were analyzed by SDS-PAGE and subjected to immunoblot analysis.

### Immunoblot analysis

Cell lysates were generated using NP-40 Alternative Lysis buffer, as described above, when cells reached ~75% confluence. In treatment cases, untreated cells or cells treated following a 24 h incubation with indicated agents were lysed as described above. Total protein concentration was determined using the Micro BCA Protein Assay Kit (Thermo Scientific, Rockford, IL, USA), and 30 *μ*g lysate was loaded onto 10% SDS-PAGE gels, resolved and transferred to PVDF membrane (Bio-Rad). Following incubation with 5% milk/0.1% Tween 20 (Thermo Fisher)/TBS blocking buffer, membranes were subjected to immunoblotting. Western blots were incubated with Super Signal West Pico Chemiluminescent Substrate (Thermo Scientific) or Super Signal West Dura Chemiluminescent Substrate (Thermo Scientific) exposed to film, and the film was developed.

### Quantitative PCR

Cells were seeded into wells of a 6-well plate (2 × 10^6^ cells/well) and allowed to adhere for ~16 h at 37 °C before the addition of agents. Media were replaced with 1 ml fresh media containing treatment or vehicle, and cells were incubated at 37 °C for 48 h. Media were aspirated, and cells were washed with PBS and incubated with 0.5 ml trypsin for several minutes at 37 °C. An equal volume of media were added to each well and cells were transferred to Eppendorf tubes and centrifuged at 1000 × *g* for 5 min. The supernatant was aspirated and RNA was extracted using a NucleoSpin RNA Kit (Macherey-Nagel, Bethlehem, PA, USA) according to the manufacturer's instructions. RNA concentration was determined by measuring *A*_260_, and SuperScript III First-Strand Synthesis System for RT-PCR (Invitrogen) was used to synthesize cDNA from 1 *μ*g RNA. Quantitative PCR was performed using a 1 : 10 dilution of generated cDNA, primers specific for human actin and TNF*α*, LightCycler 480 SYBR Green I Master (Roche, South San Francisco, CA, USA) and a LightCycler 480 Instrument (Roche).

### Statistics

Data highlighted as significant, as indicated with an asterisk (*) in each figure, were determined in all cases by Student's *T*-test. The threshold for significance in all cases was set with a probability of <0.05.

## Abbreviations

IAP: inhibitor of apoptosis protein
IAPa or I: IAP antagonist
zVAD or Z: pancaspase inhibitor; Z-Val-Ala-DL-Asp-FMKIZ, IAPa plus zVAD
TNF*α*: tumor necrosis factor-*α*
RIPK3: receptor-interacting serine–threonine kinase-3
RIPK1: receptor-interacting serine–threonine kinase-1
HGSOC: high-grade serous ovarian cancer
c-IAP1/2: cellular inhibitor of apoptosis protein 1/2
TRAF2/5: TNF receptor-associated factor 2/5
NF-*κ*B: nuclear factor-*κ*-light-chain-enhancer of activated B cells
FADD: Fas-associated protein with death domain
XIAP: X-linked inhibitor of apoptosis
ML-IAP: a member of IAP family, containing a single copy of a baculovirus IAP repeat (BIR) as well as a RING-type zinc-finger domain
MLKL: mixed lineage kinase domain-like
