# Supplementary material for: Triggering necroptosis in cisplatin and IAP antagonist-resistant ovarian carcinoma
Source: Cell Death Dis. 2014 Oct 30;5(10):e1496–. doi: 10.1038/cddis.2014.448 (PMC4237265; doi:10.1038/cddis.2014.448)
Supplement: Supplementary Figures [file cddis2014448x1.pdf]

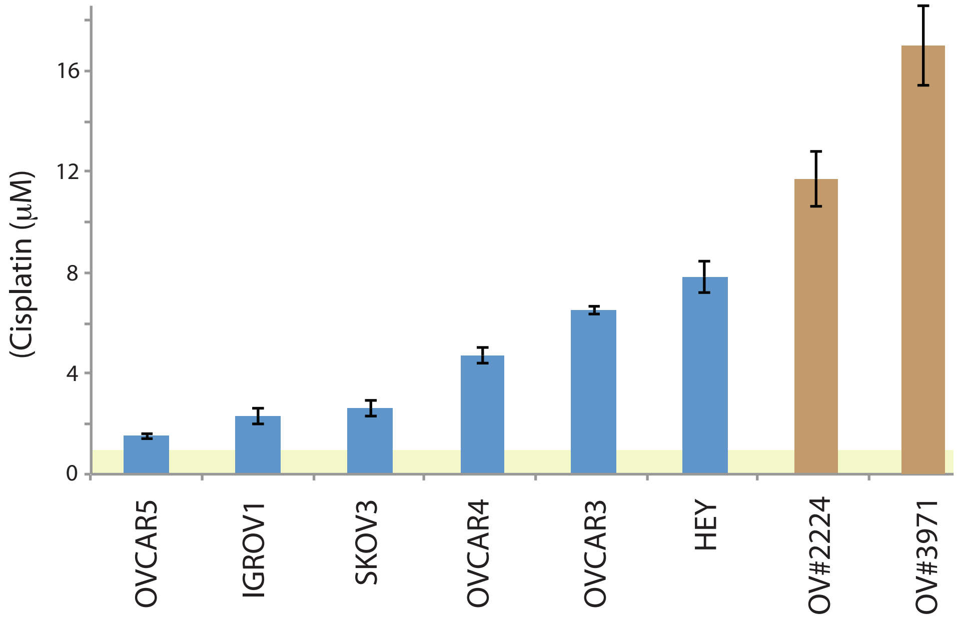

Figure 1a: Cisplatin resistance of Ovarian cells used: The IC_{50} for each line and patient xenograft was determined by incubating with cisplatin at concentrations ranging from 0.1μM to 50μM for 48h.

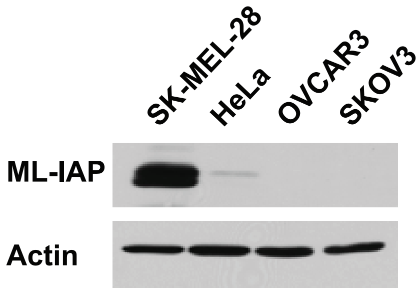

Figure 1b: Ovarian Cancer cells lack expression of the melanoma E3 ligase ML-IAP: Immunoblot analysis of the expression of ML-IAP in SK-MEL-28 (melanoma) cells, Hela cells, and apoptotic (SKOV3) and necroptotic (OVCAR3) ovarian cancer cell lines.
